# Maternal Predator Odor Exposure in Mice Programs Adult Offspring Social Behavior and Increases Stress-Induced Behaviors in Semi-Naturalistic and Commonly-Used Laboratory Tasks

**DOI:** 10.3389/fnbeh.2018.00136

**Published:** 2018-07-11

**Authors:** Sophie St-Cyr, Sameera Abuaish, Richard L. Spinieli, Patrick O. McGowan

**Affiliations:** ^1^Center for Environmental Epigenetics and Development, Department of Biological Sciences and Department of Cell and Systems Biology, University of Toronto Scarborough, Toronto, ON, Canada; ^2^Psychobiology Graduate Program, School of Philosophy, Science and Literature, University of São Paulo, São Paulo, Brazil; ^3^Department of Psychology, University of Toronto, Toronto, ON, Canada; ^4^Department of Physiology, University of Toronto, Toronto, ON, Canada

**Keywords:** stress response, social behavior, predator odor, periventricular nucleus, oxytocin, maternal programming, mineralocorticoid receptor, foraging

## Abstract

Maternal stress has a profound impact on the long-term behavioral phenotype of offspring, including behavioral responses to stressful and social situations. In this study, we examined the effects of maternal exposure to predator odor, an ethologically relevant psychogenic stressor, on stress-induced behaviors in both semi-naturalistic and laboratory-based situations. Adult C57BL/6 mice offspring of dams exposed to predator odor during the last half of pregnancy showed increased anti-predatory behavior, more cautious foraging behavior and, in the elevated plus maze, avoidance of elevated open areas and elevated open areas following restraint stress challenge. These offspring also exhibited alterations in social behavior including reduced free interaction and increased initial investigation despite normal social recognition. These changes in behavior were associated with increased transcript abundance of corticotropin-releasing factor, mineralocorticoid receptor and oxytocin (Oxt) in the periventricular nucleus of the hypothalamus. Taken together, the findings are consistent with a long-term increase in ethologically-relevant behavioral and neural responses to stress in male and female offspring as a function of maternal predator odor exposure.

## Introduction

Particularly early in life, mothers mediate information about the environment their offspring are likely to experience. This information is crucial in programming the long-term phenotype of the offspring, including responses to stress at the behavioral, physiological and transcript abundance level in stress-sensitive genes (Weaver et al., 2004; Mommer and Bell, 2014; St-Cyr and McGowan, 2015; St-Cyr et al., 2017; McGowan and Matthews, 2018). Studies in naturalistic conditions indicate that fitness and life history strategies of the offspring can be affected by these changes in phenotype (Sheriff et al., 2009; Zanette et al., 2011). For example, environmental stressors that animals have experienced over evolutionary times have been proposed to lead to adaptive programming of offspring by optimizing behavioral responses to the threat of predation (Love et al., 2013).

In naturalistic settings, predator cues generally elicit behavioral inhibition, including immobility, hiding and risk-assessment behaviors in prey animals (Dielenberg et al., 2001). Changes in foraging patterns have been observed in several species in their natural habitat in relation to the density of predators present in the environment (Lima and Dill, 1990; Creel et al., 2009). The perception of risk is also exacerbated. In several species, prey exposed to predator cues initiate flight at a greater distance from a predator in their natural habitat (Stankowich and Blumstein, 2005). Species ranging from invertebrates to fish, amphibians and mammals in a variety of habitats show decreases in general activity in the presence of predators or predator cues (Sih et al., 1992; Chivers and Smith, 1994; Hill and Lodge, 1994; Korpimaki et al., 1996; Lima, 1998).

In laboratory settings, behavioral changes in response to the presence of predators or predator cues have been primarily assessed in prey animals using tests of exploratory behavior. For example, male mice exposed to a rat, a cat or cat feces increase their avoidance of the area near the odor and limit their exploration of open elevated areas and risk assessment in the elevated-plus maze (EPM; Calvo-Torrent et al., 1999; Belzung et al., 2001; Adamec et al., 2004). Rodents exposed to 2,5-dihydro-2,4,5-trimethylthiazoline (a constituent of fox urine/feces), dog feces, a cat collar, or anal gland secretions from dogs and coyotes show a longer latency to emerge from a shelter, decreased motor activity and visits to the center of an open field, and increased freezing and vigilance behavior (Dielenberg et al., 2001; Fendt et al., 2005). Acute or repeated exposure of male rats to a cat, cat odor, or a ferret reduces their exploratory behavior (Plata-Salamán et al., 2000; Armario et al., 2008). Therefore, the impact of the presence of predators or predator cues leads to measurable changes in exploration detected both in laboratory and in natural environments.

Few studies have examined the impact of predation risk on social behavior. In fish, gravid stickleback females exposed to predation risk produced juvenile offspring showing tighter social shoaling behavior, an anti-predatory defense (Giesing et al., 2011). Studies of rats exposed to a cat or cat odor found evidence of decreased social investigation and interaction (Zangrossi and File, 1992; Blundell et al., 2005; Adamec et al., 2007).

The paraventricular nucleus (PVN) of the hypothalamus exerts prime neuroregulatory control of responses to stress and social behavior, although its role in the context of exposure to cues that predict predation threat has been suggested (Takahashi, 2014; Kondoh et al., 2016). The PVN integrates limbic and other upstream inputs to the hypothalamic-pituitary-adrenal (HPA) endocrine stress response system. We previously found that offspring from predator odor-exposed dams (PO) exhibit increased stress-related behaviors associated with epigenetic modifications and altered transcript abundance of stress-related genes in the hippocampus and amygdala (St-Cyr and McGowan, 2015; St-Cyr et al., 2017). The PVN initiates the endocrine response to stress through corticotropin-releasing factor (*Crf*) and arginine vasopressin (*Avp*) release into the portal circulation, reaching the pituitary. The pituitary then synthesizes and secretes adrenocorticotropic hormone (ACTH), which induces adrenal release of glucocorticoids, mainly corticosterone in rodents. In turn, glucocorticoids bind to mineralocorticoid receptor (*Nr3c2*) and glucocorticoid receptor (*Nr3c1*) in a number of brain areas, including the PVN, regulating the further release of glucocorticoids (Chrousos, 1998; McGowan and Matthews, 2018). Similarly, the PVN is a primary region involved in the synthesis of oxytocin (*Oxt*). *Oxt* transcript abundance in the PVN is decreased by prenatal restraint stress and postnatally following an immobilization stress, and reduced levels of *Oxt* are associated with decreased social affiliation, social memory and social interaction (de Souza et al., 2013; Muroy et al., 2016). These interactions are thought to occur via binding of *Oxt* to the widely distributed *Oxt* receptor (*Oxtr)*, activating limbic brain regions including the amygdala (Gimpl and Fahrenholz, 2001).

The objectives of this study were to examine behavioral responses in stressful laboratory-based tasks and semi-naturalistic conditions in the adult offspring of dams exposed to predator odor in pregnancy. The laboratory-based tasks consisted of the EPM, commonly-used to assess stress-related behavior (Carobrez and Bertoglio, 2005) and EPM following a 15 min restraint stress challenge (modified from Albonetti and Farabollini, 1992; Zimprich et al., 2014). The naturalistic tests consisted of the standardized Mouse Defense Test Battery (MDTB; Griebel and Beeské, 2011) and Foraging Test (FT; modified from Troxell-Smith et al., 2016). The MDTB is a standardized test used to evaluate defensive behaviors in mice in response to a threatening predator. Rats are predators of mice, as up to 77% of wild and domestic laboratory rats predate on mice (Karli, 1956; Galef, 1970). Mice show aversion to brightly lit compartments following rat odor exposure (Hebb et al., 2003). Further, laboratory rats elicit flight and risk assessment behaviors in mice, likely needed to gather information about the threat source (Griebel and Beeské, 2011). The FT was developed to assess foraging, a natural behavior exhibited by mice in the wild to find and gather food. We selected these semi-naturalistic tasks in part because they are amenable to scalable implementation in a laboratory setting. We also examined social behavior, including social investigation, social recognition and interaction. We hypothesized that maternal predator odor exposure would program adaptive behavioral responses in adult offspring. We further hypothesized that these behavioral responses would involve changes in transcript abundance in the PVN of genes involved in the stress response and social behavior.

## Materials and Methods

### Animal Housing, Breeding and Predator Odor Exposure in Pregnant Dams

Adult C57BL/6 mice were ordered from Charles River Canada (St. Constant, QC, Canada), housed in same-sexed groups (3–5 per cage) and maintained on a 12:12 h light-dark cycle (lights on at 7:00 AM) with *ad libitum* access to food and water. This study was carried out in accordance with the recommendations of the regulatory standards of the Local Animal Care Committee at the University of Toronto in Scarborough and were in accordance with the guidelines of the Canadian Council on Animal Care. The protocol was approved by the Local Animal Care Committee at the University of Toronto in Scarborough.

**Figure 1:**
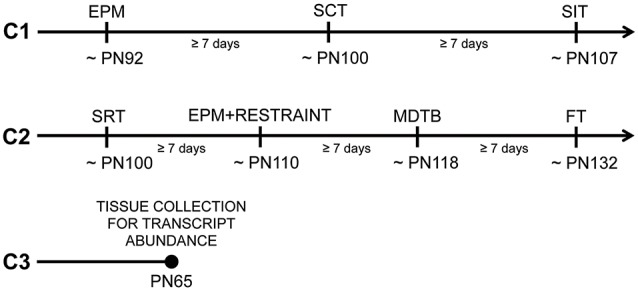
Timeline and subsets of tested offspring from predator odor and water exposed dams during pregnancy. Cohort 1 (C1) mice were tested in the following order: the elevated-plus maze (EPM), the social choice test (SCT) and the social interaction test (SIT). Cohort 2 (C2) mice were tested in the following order: the social recognition test (SRT), the EPM following 15 min of restraint (EPM + RESTRAINT), the mouse defense test battery (MDTB) and the foraging test (FT). Cohort 3 (C3) mice were sacrificed without being behaviorally tested for transcript abundance measurements in the paraventricular nucleus (PVN) of the hypothalamus and amygdala. PN, Postnatal day.

Two females were housed with one male overnight for breeding. Females were checked the next morning for sperm plugs indicating gestational day 0. Pregnant females were singly housed on gestational day 10 and weighed every day throughout the pregnancy. Cohort 1 was composed of 19 females that gave birth to eight control (C) litters and 11 PO litters. Cohort 2 was composed of 24 females that gave birth to 12 C litters and 12 PO litters. Littermates from 10 C litters and nine PO litters in cohort 2 comprised Cohort 3 (Figure 1).

The procedures for predator odor exposure in pregnant dams are described in detail elsewhere (St-Cyr and McGowan, 2015). Briefly, pregnant dams were exposed every day to a randomized predator odor (coyote urine, bobcat urine or 5 μL of a 1:5000 dilution of 2,3,5-Trimethyl-3-thiazoline) at random times during the light phase of the light:dark cycle, or to control distilled water at 4 PM each day between gestational days 11–18.

### General Behavioral Testing Procedures in Adult Offspring

Behavioral testing began when mice were adults (postnatal day, PN 86). Mice were handled (3 min/day) for seven consecutive days prior to testing to habituate them to the experimenter. Subsets of male and female adult offspring (1–2/sex/litter; Cohort 1) were assigned to a set of social and anxiety-like behavior tests in the following order: EPM, social choice test (SCT) and social interaction test (SIT). An independent cohort of mice (Cohort 2) was used to examine social and anxiety-like behavior in the following order: social recognition test (SRT), restraint followed by EPM, MDTB and FT. The order of testing for each behavioral test was pseudo-randomized between maternal predator odor exposure groups and sexes. After each behavioral test, the testing apparatus was cleaned with 70% ethanol. There was at least a week time interval between each test. A final independent set of untested adult mice (PN65, Cohort 3) was used for transcript abundance measurements (control males [C-M]: *n* = 6, control females [C-F]: *n* = 6, male offspring from predator odor exposed dams [PO-M]: *n* = 6, female offspring from predator odor exposed dams [PO-F]: *n* = 6). The testing timeline and cohorts of animals are shown in Figure 1.

### Elevated-Plus Maze and Elevated-Plus Maze Following 15 min of Restraint

The 15 min EPM test consisted of a platform with two open and two closed arms (35.5 cm × 5 cm) around a central zone (5 cm × 5 cm) elevated 80 cm above ground under red lighting. The position and activity level of each mouse (C-M: *n* = 16, C-F: *n* = 14, PO-M: *n* = 20, PO-F: *n* = 20) was measured continuously using EthoVision XT 10 (Noldus, Toronto, ON, Canada).

The 15-min test of restraint followed by the EPM consisted of 15 min of restraint in a transparent pierced bag (Wilton) under red lighting followed by 5 min in an empty cage (19 cm × 39 cm). This 5-min period was meant to allow the mouse to self-groom (Zimprich et al., 2014) to avoid confounding the animals’ exploratory behavior in the EPM. In addition, it is known that blood corticosterone levels peak 20 min after the stress onset (Spiga et al., 2014), at which time the EPM test started. The EPM test lasted 15 min in the same apparatus and method described above (C-M: *n* = 14, C-F: *n* = 11, PO-M: *n* = 13 and PO-F: *n* = 10).

### Mouse Defense Test Battery

The MDTB protocol implemented in this study has been described in detail previously (Griebel and Beeské, 2011). Briefly, the tested mouse (C-M: *n* = 13, C-F: *n* = 11, PO-M: *n* = 11 and PO-F: *n* = 10) was exposed to a rat puppet (30 cm long × 13 cm wide × 10 cm high, Folkmanis) handled by an experimenter and housed in a soiled male rat cage. Rats are a predator of mice (Karli, 1956; Galef, 1970) and rat odor cues are known to be aversive (Merali et al., 2003). Mice were exposed to the rat puppet in an oval runway (40 cm wide × 30 cm high × 2 times 200 cm straight segments joined by two 40 cm curved segments and separated by a median wall, see Griebel and Beeské, 2011).

Activity was first recorded over a 3-min pretest habituation period (without the rat puppet) during which risk assessment and escaping behaviors were measured. This period was followed by a predator avoidance test, where the rat puppet was introduced at one end of the runway and accelerated towards the mouse at a 50 cm/s speed until the subject ran away or was brought into contact with it. This test was repeated five times. The distance to the predator at flight initiation was measured.

The chase/flight test consisted of a chase at 200 cm/s over 15 m, with the rat staying at a distance of 20 cm from the test mouse. The time to run 15 m, the speed and the number of stops, and stops with orientation towards the predator during the flight were measured. For the straight alley test, the subject was constrained between partitions placed in the runway 60 cm apart, the rat puppet was introduced at one end of the alley for 30 s and behaviors (immobility time, closest distance to the predator and number of approach/withdrawal behavior) were recorded.

The forced contact test consisted of three, 1-s approaches of the rat puppet within the straight alley. The following behaviors were measured: biting, vocalizations, aggressive upright posture and jump attacks. Finally, post-test activity was measured for 3 min in the full runway. Mouse position and activity level were measured continuously using EthoVision XT 10 (Noldus, Toronto, ON, Canada).

### Foraging Test

The giving-up density (GUD) in a finite food patch is defined as a measure of the quitting harvest rate (i.e., food left on a food patch) in different environmental situations, which provides a means to titrate or balance food and safety (Brown and Kotler, 2004). In natural animal populations, predator presence or cues and foraging patch cover density (overhead protection) are known to determine the GUD (Brown and Kotler, 2004). Here, we tested the effect of rat odor on the food left in both concealed and exposed finite food patches. The protocol used in this study was a modification of the one used by Troxell-Smith et al. (2016), which enabled us to perform a higher-throughput evaluation of foraging behavior in a laboratory setting.

**Figure 2:**
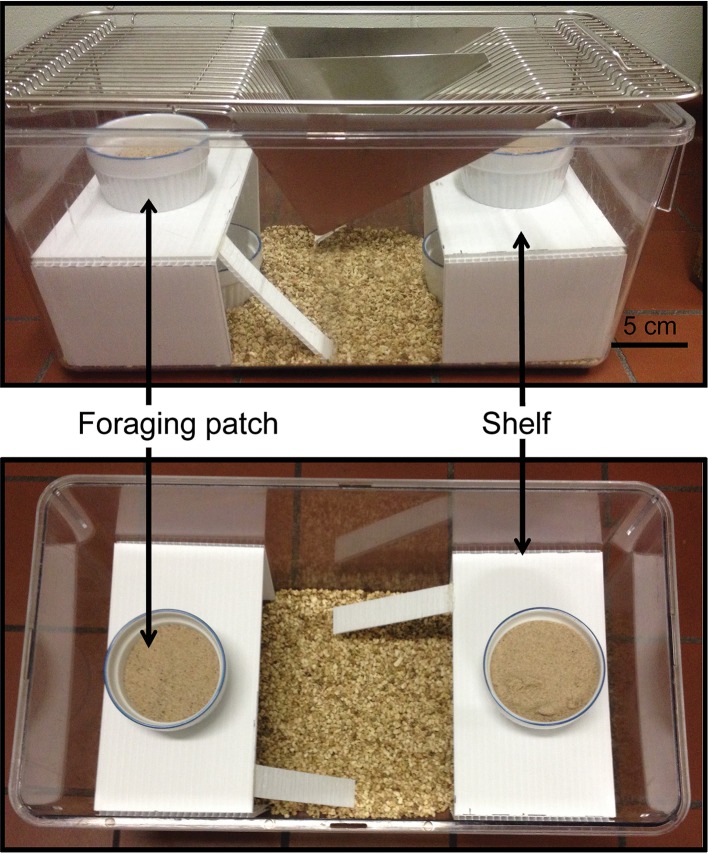
FT apparatus. Two replicate foraging patches containing sunflower seeds mixed with sand were exposed on top of the shelves and two replicate concealed patches were placed under the shelves to examine foraging risk in finite foraging patches.

Mice (C-M: *n* = 13, C-F: *n* = 11, PO-M: *n* = 12 and PO-F: *n* = 10) were tested in 45 cm long × 24 cm wide × 20.5 cm high transparent cages. The cages contained bedding, four foraging patches, a dish with water and two 10 cm high platforms with access ramps (Figure 2). Two patches were exposed on the top of the platforms and two were concealed underneath the platforms. The foraging patches (7.5 cm diameter) contained a mix of 8 g of sand and 3.5 g of hulled sunflower seeds. Prior to testing, mice were exposed to hulled sunflower seeds in their home cage for four consecutive days (habituation days 1–4). On the fifth day, mice were exposed to a small novel cage (15 cm × 33 cm) with a shallow foraging patch (1.5 cm deep petri dish) with a high concentration of buried and exposed sunflower seeds from 9 AM to 12 PM while on day 6, the same protocol was applied using a small ceramic baking dish (a ramequin). These habituation phases were used to ensure that the mice would consume the bait and learn to dig to access the food in the foraging patches. On the day of testing, eight cages were laid down in four rows of two cages, separated by white partitions to prevent visual interactions between the animals. Soiled rat cages separated the four rows. Individual mice were placed in the testing cages allowing the testing of eight animals at a time. Mice were food-deprived for 17 h prior to the test and housed overnight in the testing room for habituation. The test lasted 6 h, from 9 AM to 3 PM. The GUD was measured for each patch following the test by weighing the uneaten seeds left in the food patch. The ratio of the GUD from the top exposed patches to the bottom concealed patches was calculated as: (GUD covered patch 1 + GUD covered patch 2/total initial food)/(GUD concealed patch 3 + GUD concealed patch 4/total initial food).

### Social Choice, Social Recognition and Social Interaction Tests

The SCT consisted of an arena divided into a center neutral zone between two choice zones (15 cm × 24 cm each), placed in a dimly lit room (33.7 lux). The choice zones contained a wire cup (8.5 cm diameter) enclosing a familiar mouse (same home cage as the focal mouse) and an unfamiliar mouse (different litter and home cage than the focal mouse). The focal mouse was placed in the center of the arena and the frequency of entering and time spent in each choice zones and distance traveled were measured over 10 min using EthoVision XT 10 (Noldus, Toronto, ON, Canada).

The SRT took place in the same arena as the SCT and consisted of four consecutive 5-min exposures to an unknown juvenile conspecific (PN33 [PN25–40]; juvenile #1) followed by a 5-min exposure to an unknown juvenile conspecific (juvenile #2), with inter-trial intervals of 10 min on the first day. On day 2, juvenile #1 was presented once and, after a 10-min inter-trial interval, juvenile #3 was exposed for the first time. Each trial was 5 min long. Juvenile CD-1 mice with a maximum of 20% weight difference with each other were used as unknown conspecifics. An initial 6-min baseline activity trial was performed on day 1 prior any unknown animal presentation. Measurements included time spent in the juvenile zone, difference in time spent in the juvenile zone across encounters, and distance traveled during the activity trial and the sociality trials (C-M: *n* = 7, C-F: *n* = 7, PO-M: *n* = 13 and PO-F: *n* = 14) measured continuously using EthoVision XT 10 (Noldus, Toronto, Canada).

The SIT (C-M: *n* = 14, C-F: *n* = 10, PO-M: *n* = 16 and PO-F: *n* = 20) took place in a Plexiglas square arena (38.5 × 38.5 cm). Two unfamiliar mice from the same maternal predator odor exposure group, sex and with a similar weight (±5%) were placed simultaneously in the arena for 15 min. Focal interactions consisting of displacement behavior, offensive and defensive behaviors, sniffing, following, jumping on, crawling under, escaping and approaching were coded continuously using Observer XT 8.5 for 5 min, based on a protocol developed by Grant and Mackintosh (1963). Aversive and aggressive interactions consisted of displacement, offensive, defensive and escaping behaviors.

### Tissue Preparation and Nucleotide Extraction

Mice were sacrificed by CO_2_ inhalation followed by decapitation on PN65. Whole brains were flash frozen in isopentane on dry ice and stored at −80°C. The entire PVN (Bregma −0.70 mm to −1.06 mm) and amygdala (Bregma −0.58 mm to −1.94 mm) were dissected from 50 μm cryosections with a research cryostat (Leica CM3050 S, Leica Biosystems) using stereotaxic coordinates (Franklin and Paxinos, 1997). RNA was extracted using the Epicentre MasterPure Complete RNA purification kit (Lucigen, Cat. MC85200). RNA was converted to complementary DNA using a High Capacity cDNA Conversion Kit (Applied BioSystems, Cat. 4368814). Nucleotide quantification and purity were assessed with a spectrophotometer (Nanodrop ND-2000C, Thermo Scientific).

### Transcript Abundance Analysis by qRT-PCR

Gene transcript abundance in the PVN and amygdala were quantified using a StepOne Plus real-time thermocycler and Fast SYBR Green PCR master mix (Applied Biosystems, Life Technologies, Carlsbad, CA, USA). The transcript abundance of genes of interest were corrected using the geometric mean of four housekeeping genes (18S Ribosomal RNA (*18s*), Actin Beta (*Actinb*), Glyceraldehyde 3-Phosphate Dehydrogenase (*Gapdh*) and 14-3-3 protein zeta/delta (*Ywhaz*)) in the PVN as this combination of housekeeping genes showed the least variability for this tissue, and *Ywhaz* in the amygdala (St-Cyr and McGowan, 2015; St-Cyr et al., 2017). The genes investigated in the PVN were *Avp*, *Crf*, *Oxt*, *Nr3c1* and *Nr3c2*, while *Oxtr* transcript abundance was measured in the amygdala. Primers were designed using sequence information from GeneBank at the National Center for Biotechnology Information (NCBI)^1^ and Ensembl^2^ (Table 1). A standard curve was generated with 11 or 12 serial dilutions of a mixture of complementary DNA from all offspring where transcript abundance was measured. Quantification was carried out in triplicate, and the average relative transcript abundance for each sample was used for analysis.

**Table 1:** Mouse primer sequences.

Gene	Forward primer (5′-3′)	Reverse primer (5′-3′)
*18s*	CCCTGAGAAGTTCCAGCACA	GTGATCACTCGCTCCACCTC
*Avp*	TCGCCAGGATGCTCAACAC	TTGGTCCGAAGCAGCGTC
*Actinb*	TTTGAGACCTTCAACACCCC	ATAGCTCTTCTCCAGGGAGG
*Crf*	GCAGCCCTTGAATTTCTTGCA	TCTTCACCCATGCGGATCAG
*Gapdh*	CCTGCACCACCAACTGCTTA	CGTTCAGCTCTGGGATGACC
*Nr3c1*	AACTGGAATAGGTGCCAAGG	GAGGAGAACTCACATCTGGT
*Nr3c2*	GAAGAGCCCCTCTGTTTGCAG	TCCTTGAGTGATGGGACTGTG
*Oxt*	GCTGCCAGGAGGAGAACTAC	CTCCCGAGAAGGCAGACTCAG
*Oxtr*	GCACGGGTCAGTAGTGTCAA	GCATGGCAATGATGAAGGCA
*Ywhaz*	TTGAGCAGAAGACGGAAGGT	GAAGCATTGGGGATCAAGAA

*18s*, 18S Ribosomal RNA; *Avp*, Arginine Vasopressin;* Actinb*, Actin Beta; *Crf*, Corticotropin-Releasing Factor; *Gapdh*, Glyceraldehyde 3-Phosphate Dehydrogenase; *Nr3c1*, Glucocorticoid receptor; *Nr3c2*, Mineralocorticoid receptor; *Oxt*, Oxytocin; *Oxtr*, Oxytocin Receptor; *Ywhaz*, 14-3-3 protein zeta/delta.

### Statistical Analysis

Statistical analyses were carried out using SPSS (IBM). Data were tested for normality using the Shapiro-Wilk test and were log transformed, log plus 1 transformed when zeros were present in the distribution, and fraction transformed in that order to achieve normality whenever possible. With normal data, a General Mixed Model (GLM) was used, whereas when the data could not be normalized, a two-tailed Mann-Whitney Test or Linear Mixed Model (LMM) was performed. Maternal predator odor exposure and/or sex were examined as main effects, with litter ID, body weight and/or distance traveled, as applicable, as random factors. Effect sizes were calculated using the partial eta-squared ($\eta_{p}^{2}$), with *η*^2^ ≥ 0.14 indicating large effect sizes (Levine and Hullett, 2002), or the Cohen’s d or d_r_ (calculated with the residuals in the LMM), with *d* ≥ 0.5 indicating moderate and *d* ≥ 0.8 indicating large effect sizes (Rice and Harris, 2005; Rouder et al., 2012). Bonferroni corrected tests were performed for *post hoc* comparisons. Effects were considered statistically significant at *P* ≤ 0.05 with all reported effects being of moderate or large effect size (St-Cyr et al., 2017, 2018).

## Results

### Elevated-Plus Maze

**Figure 3:**
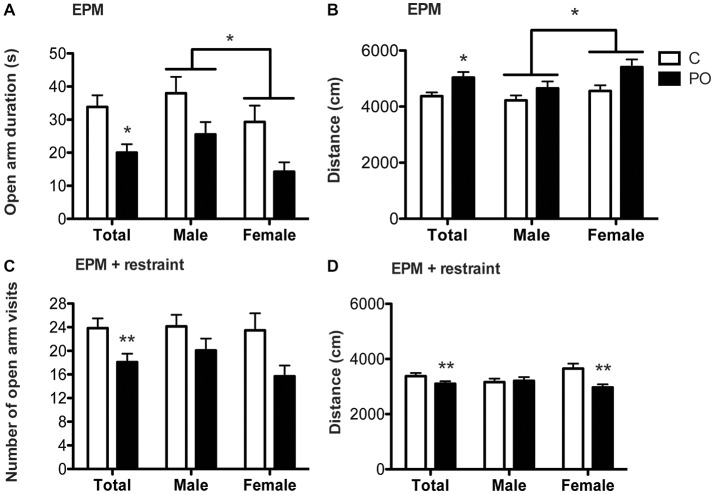
Adult offspring from predator odor-exposed dams (PO) show increased stress-related behavior in the EPM and the EPM following restraint tests in adulthood. In the EPM, PO offspring showed decreased time spent in the open arm **(A)** and increased levels of activity **(B)** compared to control **(C)** offspring. Females also traveled a greater distance than males in the EPM. In the EPM following restraint, PO offspring showed decreased visits in the open arms **(C)** as well as general hypoactivity, especially in female PO offspring **(D)**. Data are average ± standard error of the mean. Connected bars: main sex effect *P ≤ 0.05; main maternal predator odor exposure effect and within sex maternal predator odor exposure effect *P ≤ 0.05, **P ≤ 0.01.

In the EPM, PO offspring spent less time in the open arm than control offspring (*F*_(1,64)_ = 5.094, *P* = 0.03, *d_r_* = 0.6; Figure 3A) and males spent more time in the open arm than females (*F*_(1,63)_ = 5.055, *P* = 0.03, *d_r_* = 1.0). The distance traveled was greater in PO offspring than in control offspring (*F*_(1,64)_ = 5.094, *P* = 0.03, *d_r_* = 0.7; Figure 3B) and was greater in females compared to males (*F*_(1,63)_ = 5.055, *P* = 0.03, *d_r_* = 0.5) over the 15 min of the test.

In the EPM following restraint, PO offspring decreased the number of visits to the open arm compared to control offspring (*F*_(1,46)_ = 5.531, *P* = 0.01, *d* = 2.3; Figure 3C). PO offspring also decreased the distance traveled over the 15 min of the test when compared to control offspring (*F*_(1,42)_ = 7.262, *P* = 0.01, *d_r_* = 0.6). Additionally, the maternal predator odor exposure influenced the distance traveled in a sex-specific manner (Maternal predator odor exposure × sex interaction: *F*_(1,42)_ = 6.009, *P* = 0.02, *d_r_* = 3.7; Figure 3D) with female PO offspring showing suppressed activity as measured by the distance travelled compared to control offspring (*F*_(1,19)_ = 13.119, *P* = 0.002, *d_r_* = 1.5; Figure 3D) while males showed no such difference (*P* > 0.05).

### Mouse Defense Test Battery

**Figure 4:**
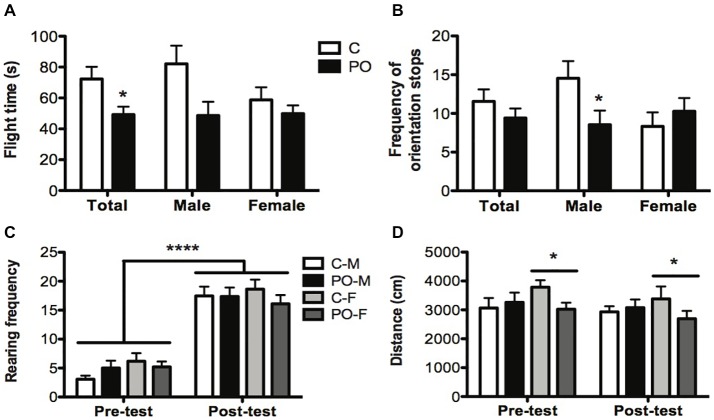
Adult offspring from predator odor-exposed dams (PO) show increased anti-predatory behavior in the MDTB when compared to control (C) offspring. In the chase/flight test, PO offspring showed a decrease in flight time (time taken to run 15 m; **A**) with a decrease in the number of stops with orientation towards the rat in male PO offspring compared to male control offspring **(B)**. All tested groups showed an increase in post-test rearing **(C)** and female (F) PO offspring showed a decrease in overall activity compared to female control offspring **(D)**. Data are average ± standard error of the mean. Large connected bars: main time (pre- and post-test) effect ****P ≤ 0.0001. Connected bars: main female maternal predator odor exposure effect *P ≤ 0.05; main maternal predator odor exposure effect and within male maternal predator odor exposure effect *P ≤ 0.05.

In the MDTB, the overall flight time from the rat puppet (time taken to run 15 m) was shorter in PO offspring when compared to control offspring (*F*_(1,29)_ = 4.552, *P* = 0.04, *d_r_* = 0.9; Figure 4A). The associated frequency of stops to orient towards the rat showed a sex-specific effect of maternal predator odor exposure (Maternal predator odor exposure × sex interaction: *F*_(1,33)_ = 4.48, *P* = 0.04, *d_r_* = 3.6). Male PO offspring showed a decreased number of orientation stops when compared to male control offspring (*F*_(1,19)_ = 18.754, *P* = 0.02, *d_r_* = 1.6; Figure 4B) while females showed no such difference (*P* > 0.05). There were no other maternal predator odor exposure differences in the MDTB predator avoidance, straight alley or forced contact tests (*P*s > 0.05).

Between the initial habituation and final activity trial, mice exhibited an overall increase in the frequency of rearing (*F*_(1,43)_ = 218.709, *P* < 0.0001, *d_r_* = 2.9; Figure 4C), a risk assessment behavior, and wall climbing (*F*_(1,43)_ = 9.008, *P* = 0.004, *d_r_* = 0.7), an escape behavior. These increases occurred in both maternal predator odor exposure groups. The distance traveled did not vary before and after the predator exposure trial (*P* > 0.05), but distance was influenced by the maternal predator odor exposure differently in each sex (Maternal predator odor exposure × sex interaction: *F*_(1,41)_ = 3.801, *P* = 0.05, *d_r_* = 3.6) as only female PO offspring showed an overall decrease in distance traveled compared to control female mice (*F*_(1,19)_ = 4.407, *P* = 0.05, *d_r_* = 0.7; Figure 4D).

### Foraging Test

**Figure 5:**
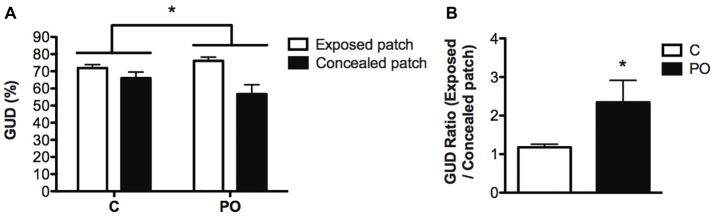
Adult offspring of predator odor-exposed dams (PO) forage more in concealed than in exposed patches when compared to control (C) adult offspring. PO offspring showed modifications in the percentage of giving-up density (GUD) in the top and bottom foraging patches **(A)** and an increase in the ratio of giving up density (GUD) between exposed and concealed patches **(B)**, indicating that they forage more intensively in concealed patches compared to control mice. Data are average ± standard error of the mean. Maternal predator odor exposure effect *P ≤ 0.05.

In natural animal populations, predators or the presence of predation cues and exposed foraging patches (exposure) are typically aversive and increase the GUD (Brown and Kotler, 2004). We therefore reasoned that PO offspring would show a higher GUD in risky exposed patches compared to concealed protected patches. Maternal predator odor exposure impacted food consumption in the differentially covered patches (Maternal predator odor exposure × patch interaction: *F*_(1,44)_ = 4.27, *P* = 0.05, *d_r_* = 3.6) with PO offspring consuming comparatively less food in the exposed patches than the covered patch relative to control offspring (Figure 5A). The ratio of the top to bottom GUD was increased significantly in PO offspring compared to control offspring (Mann–Whitney *U* = −1.935, *n*_C_ = 24, *n*_PO_ = 22, *P* = 0.05, *d* = 3.4; Figure 5B), indicating that PO offspring showed decreased time spent feeding on the exposed patches vs. the concealed patches, when compared to control offspring. When comparing the top GUD to the bottom GUD, more of the food from the concealed patches was consumed compared to the one in exposed patches in all groups (*F*_(1,44)_ = 14.564, *P* < 0.0001, *d_r_* = 0.7). PO offspring consumed the same amount of food overall as control offspring when corrected for body weight (*P* > 0.05). The offspring consumed the same amount of food in each of the replicates of concealed patches or each of the replicates of exposed patches within a trial (*P* > 0.05).

### Social Behavior

**Figure 6:**
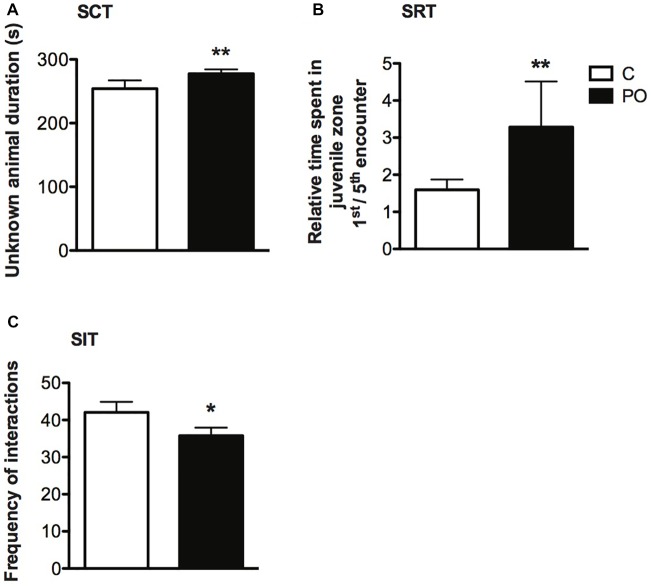
Adult offspring of predator odor-exposed dams (PO) show increased social investigation during an initial encounter with an unknown conspecific, no difference in social recognition and decreased social interactions when compared to control (C) offspring. In the SCT, PO offspring of both sexes showed increase investigation of an unknown compared to a known conspecific **(A)**. In the SRT, the ratio of the time spent during a first encounter with an unknown conspecific vs. the fifth encounter with the same mice (now a known conspecific) was higher in PO offspring of both sexes, indicating an increase in social investigation during a first encounter **(B)**. In the SIT, the total number of interactions was decreased between unknown PO offspring compared to unknown control conspecifics interacting freely for the first time **(C)**. Data are average ± standard error of the mean.Main maternal predator odor exposure effect *P ≤ 0.05, **P ≤ 0.01.

In the SCT, PO offspring explored the unknown conspecific zone for a longer duration when compared to control offspring (*F*_(1,40)_ = 9.068, *P* = 0.005, *d* = 1.0; Figure 6A). Further, PO offspring explored the unknown conspecific zone for a longer duration relative to the known conspecific zone when compared to control offspring (*F*_(1,37)_ = 7.302, *P* = 0.01, *d_r_* = 0.9). There was no difference in the distance traveled between the maternal predator odor exposure groups (*P* > 0.05).

In the SRT, time spent in the juvenile zone within encounters 1 through 5 did not differ between PO and control offspring (*P* > 0.05), indicating no difference in social recognition in PO offspring. However, maternal predator odor exposure influenced the duration spent in the juvenile zone over successive encounters (Maternal predator odor exposure × encounter interaction: *F*_(4,2207)_ = 3.553, *P* = 0.007, *d_r_* = 5.7). When examining the ratio of time spent in the juvenile zone on a first vs. a fifth encounter, PO offspring showed decreased time spent in the juvenile zone over time relative to control offspring (Mann–Whitney *U* = 340, *n*_C_ = 22, *n*_PO_ = 21, *P* = 0.003, *d* = 0.9; Figure 6B). Similarly to the SCT test, PO offspring investigated the conspecific longer during the first encounter with the unknown conspecific when compared to control offspring, but not during encounter two through five with this same conspecific (*P* > 0.05). Time spent in proximity to the juvenile decreased with time in all maternal predator odor exposure groups (Encounter effect: *F*_(4,2307)_ = 40.229, *P* < 0.0001, *d_r_* = 3.0). There was no difference in the initial activity level (distance traveled during the activity trial) between PO and control offspring (*P* > 0.05).

In the SIT, there was an overall decrease in the total number of social interactions performed (pooled displacement, offensive, defensive, sniffing, following, jumping on, crawling, escaping and approaching behaviors) in PO offspring freely interacting with an unknown conspecific compared to control offspring (Mann–Whitney *U* = 330.5, *n*_C_ = 27, *n*_PO_ = 36, *P* = 0.03 two-tailed, *d* = 0.6; Figure 6C). This difference was particularly pronounced among negative (aversive or aggressive) interactions, including displacement, offensive, defensive and escaping behaviors (Frequency: Mann–Whitney *U* = 280, *n*_C_ = 27, *n*_PO_ = 36, *P* = 0.004, *d* = 0.8; Duration: Mann–Whitney *U* = 258, *n*_C_ = 27, *n*_PO_ = 36, *P* = 0.002, *d* = 0.9). PO mice also approached the unknown conspecific less frequently than control mice (Mann–Whitney *U* = 351, *n*_C_ = 27, *n*_PO_ = 36, *P* = 0.05, *d* = 0.5).

### Transcript Abundance

**Figure 7:**
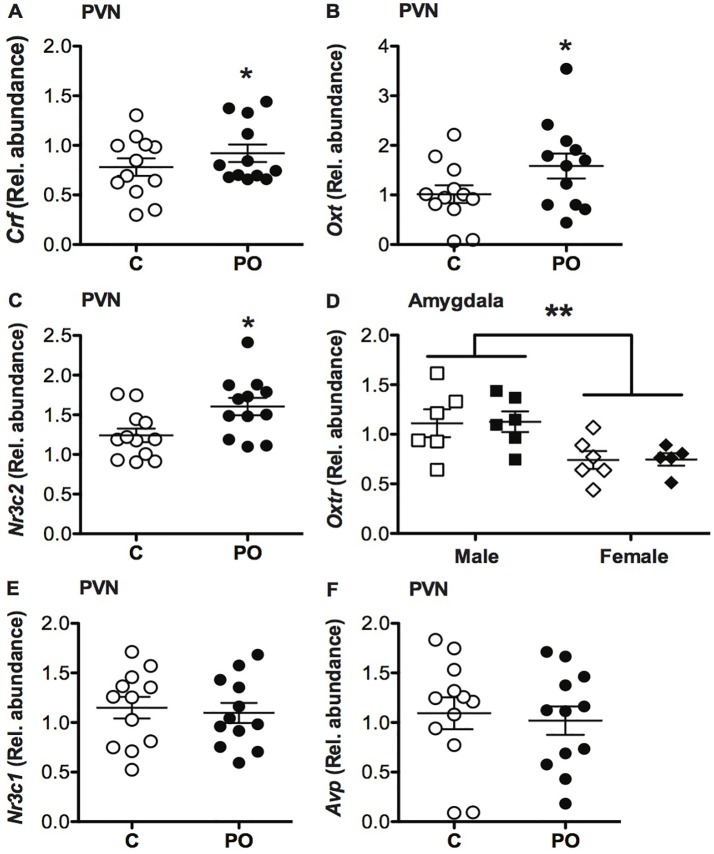
Adult offspring of predator odor-exposed dams (PO) show altered transcript abundance in the PVN of the hypothalamus when compared to control (C) adult offspring. PO offspring showed increase corticosterone-releasing factor (*Crf*; **A**), oxytocin (*Oxt*; **B**) and mineralocorticoid receptor (*Nr3c2*; **C**) transcript abundance in the PVN compared to control adult offspring. PO offspring showed similar oxt receptor (*Oxtr*) transcript abundance in the amygdala as control offspring, with higher levels of *Oxtr* in males than females **(D)**. No difference in glucocorticoid receptor (*Nr3c1*; **E**) or arginine vasopressin (*Avp*; **F**) transcript abundance in the PVN was detected between PO and control offspring. Data are average ± standard error of the mean. Main maternal predator odor exposure effect *P ≤ 0.05. Connected bars: sex effect **P < 0.01.

In the PVN, PO offspring showed increased transcript abundance of *Crf* (*F*_(1,23)_ = 5.078, *P* = 0.04, $\eta_{p}^{2}$ = 0.20; Figure 7A), *Oxt* (*F*_(1,23)_ = 4.31, *P* = 0.05, $\eta_{p}^{2}$ = 0.18; Figure 7B) and *Nr3c2* (*F*_(1,23)_ = 6.31, *P* = 0.02, $\eta_{p}^{2}$ = 0.24; Figure 7C) when compared to control offspring. No significant differences in *Nr3c1* (Figure 7E) or *Avp* (Figure 7F) transcript abundance were detected in the PVN (*P* > 0.05) and no significant sex difference was detected in transcript abundance in the PVN (*P* > 0.05).

In the amygdala, there was no difference in *Oxtr* transcript abundance between the PO and control offspring (*P* > 0.05), however, males generally had a higher *Oxtr* transcript abundance overall compared to females (*F*_(1,23)_ = 12.280, *P* = 0.002, $\eta_{p}^{2}$ = 1.55; Figure 7D).

## Discussion

In this study, we investigated whether maternal predator odor exposure could program behavioral adaptations to a high-risk environment in adult offspring. To our knowledge, this study is the first to use semi-naturalistic behavioral screening in comparison to commonly-used exploratory-based laboratory tasks to examine maternal stress effects. Overall, we discovered that PO offspring of both sexes showed an increase in stress-related behaviors in the EPM, with and without pre-exposure to restraint stress, and in foraging and anti-predatory assessments, with sex differences detected in some tasks. These behavioral changes in PO offspring were associated with increased transcript abundance of *Crf*, *Nr3c2* in the PVN, a region that exerts prime neuroregulatory control of the stress response. In addition, PO offspring explored a novel mouse for a longer period of time during a first encounter but did not show differences in social recognition and memory. Consistent with these results, transcript abundance of *Oxt*, a regulator of social behavior primarily produced in the PVN (Gimpl and Fahrenholz, 2001), was increased in the PVN of PO offspring.

Maternal endocrine state and behavior are likely contributors to the PO offspring phenotype observed in this study. We previously found that dams exposed to predator odor show increased fecal corticosterone at the end of the pregnancy (gestational day 18) as well as increased licking/grooming and decreased nest quality in the early postpartum period (St-Cyr et al., 2018). In addition, female PO rat offspring show elevated *Nr3c1* gene expression at birth in the amygdala compared to control females. This difference was no longer present in adulthood but these females rats displayed increased expression of the *Nr3c1* co-chaperone *Fkbp5* in adulthood (St-Cyr et al., 2017). These data indicate that the persistent effects of maternal PO exposure on offspring phenotype involve a complex interplay of both prenatal and postnatal effects on stress-related signaling pathways.

PO offspring of both sexes showed increased stress-related behavior in the standard EPM task (no stress challenge), with decreased time spent in open arms and increased locomotor activity. Consistent with these findings, PO offspring showed a decreased frequency of visits to the open arms in the EPM after acute stress (restraint). We also observed a reduction in activity following restraint, a hallmark effect of restraint stress (reviewed in Buynitsky and Mostofsky, 2009) including in the EPM (Padovan and Guimarães, 2000), and a drastic difference compared to the hyperactivity of the PO offspring observed in the EPM test without prior stress exposure. In females, this hypoactivity was more pronounced in PO offspring compared to control offspring, suggesting greater sensitivity to the restraint stress.

Both control and PO offspring displayed an increase in rearing and wall climbing in the MDTB at the end of the simulated rat predator exposure, constituting risk assessment and escape behavior, respectively. These results confirm the aversive nature of the exposure used in this task, as rats are known to elicit flight and risk assessment behavior in mice (Griebel and Beeské, 2011). Female PO offspring were hypoactive compared to female control offspring in the empty novel apparatus containing rat odor, a documented anti-predatory behavior (Blanchard and Blanchard, 1989; Apfelbach et al., 2005). When the simulated predator chased the mice, PO offspring took less time to flee 15 m than control offspring. PO males stopped less frequently to orient themselves toward the simulated rat, a risk-assessment behavior decreasing the efficiency of the flight response. These observations indicate that the PO offspring were more efficient in their anti-predator flight responses than control offspring.

In this study, we used a uniform rat odor to create a risky environment (Hebb et al., 2003) in exposed and concealed foraging patches. The foraging rate of prey species is determined by the interaction of several costs of foraging including predation risk (Brown and Kotler, 2004). The GUD, as measured in this study, is the amount of food left in a depletable food patch after a given foraging time and is thought to indicate a trade-off of food for safety. Consequently, although consuming the same total amount of food, the control and PO offspring distributed their foraging efforts differently across the patches. PO offspring left more food in the exposed patches compared to the concealed ones when compared to control offspring independently of their body weight. The amount of food left in a patch is known to be determined by several factors including the perceived “riskiness” of the patch, which can be altered by its degree of coverage. There is evidence of increased foraging risk or perceived risk in exposed patches when compared to concealed ones (Troxell-Smith et al., 2016), especially in a risky environment containing cues that indicate the presence of predators (Dickman, 1992; Thorson et al., 1992; Christensen and Persson, 1993; Shrader et al., 2008). Adaptive programming theory predicts an increase in aversion to a risky foraging situation (Bateson et al., 2014). It is therefore likely that PO offspring in this study favored food consumption in patches perceived to be safer with exposure to uniform predator odor. These findings suggest that our scalable version of the FT to examine foraging behavior in a laboratory setting could be included in behavioral test batteries aiming at uncovering stress-associated phenotypes.

PO offspring showed a decrease in reciprocal social interactions when exposed to an unknown conspecific in the SIT, similar to the impact of other prenatal stressors (de Souza et al., 2013; Matrisciano et al., 2013; Dong et al., 2015). We also observed similar social recognition memory in PO offspring and control offspring. Surprisingly, PO offspring showed an increase in the initial social investigation of an unknown conspecific, a robust observation made twice in different cohorts of animals. This difference was not observed in subsequent encounters with the same conspecific or in subsequent first encounters with other unknown conspecifics. The lack of social recognition memory deficit and specific increase in social investigation during the first encounter with an unknown conspecific contrasts with the results of other prenatal stressors such as chronic restraint stress, chronic variable stress and social stress (resident-intruder) that have been reported to lead to a decrease in social recognition memory and no change or a decrease in social investigation (Lee et al., 2007; Jones et al., 2010; Grundwald et al., 2016; reviewed in Kundakovic and Jaric, 2017).

It is possible that increased transcript abundance of *Crf* in the PVN detected in the PO offspring of both sexes contributed to the differences in social behaviors between PO and control offspring. In line with the initial increase in social investigation in PO offspring, male mice overexpressing *Crf* in the PVN of the hypothalamus beginning in the early postnatal period (PN 4–8) show increased social investigation during a first encounter with an unknown mouse along with normal short-term social recognition memory in adulthood (Kasahara et al., 2011). Similarly, the indirect *Crf* antagonist Methysergide impairs juvenile recognition, an effect that is reversed by the restoration of normal levels of *Crf* transcription (Heinrichs et al., 1997). Prenatal stressors including hypoxia and/or restraint lead to increased *Crf* and *Crfr1* transcript abundance in the PVN in adult mice, associated with an increase in ACTH and corticosterone levels (Fan et al., 2009) suggesting a contribution to stress-related behaviors. Interestingly, we previously found increased *Crfr1* transcript abundance in the amygdala of adult female PO offspring mice as well as increased circulating corticosterone during adult exposure to a predator odor for the first time (St-Cyr and McGowan, 2015). In previous work by our group, *Nr3c1* and *Nr3c2* transcript abundance did not differ in the PO offspring hippocampus, limiting its potential involvement in the social behavior phenotype observed in this study (St-Cyr and McGowan, 2015; St-Cyr et al., 2017). Taken together, the increase in *Crf* transcript abundance within the PVN may mediate in part the increase in stress-related behavior, initial social investigation and normal social memory in PO offspring.

PO offspring of both sexes exhibited increased *Oxt* transcript abundance within the PVN, the primary brain region of *Oxt* expression (Gimpl and Fahrenholz, 2001). These results contrast with impairments in social memory and decreased social interaction reported in adult rat offspring from dams exposed to repeated physical restraint during gestation, that show a reduction in the number of *Oxt*-positive cells in the PVN (de Souza et al., 2013). However, other evidence has indicated that increased *Oxt* mediates increased social affiliation following acute immobilization stress (Muroy et al., 2016). PO offspring had unaltered *Oxtr* transcript abundance within the amygdala. This was unexpected given previous evidence of the role of *Oxtr* in amygdala in promoting social behavior (Gimpl and Fahrenholz, 2001). The lack of difference in amygdala *Oxtr* expression, suggests that the increased social investigation observed in this study may occur through another as yet undefined mechanism. For example, increased *Oxtr* binding in the central amygdala was detected in male rats prenatally exposed to unpredictable stressors (Lee et al., 2007) where binding density is correlated with social investigation (Dumais et al., 2013). This result suggests that binding is influenced by factors beyond the level of hormone and ligand. For example, *Oxt* binds with low affinity to *Avp* receptors, which could further mediate the impacts of higher central *Oxt* expression (Russell and Brunton, 2009). Future studies should however investigate *Oxtr* transcript abundance in the olfactory bulb and cortex and amygdala sub-nuclei as these differences might be obscured in an analysis of transcript abundance in the entire amygdala. For example, the medial amygdala is necessary for olfactory-based social recognition in both males and females while preserving the olfactory non-social olfactory memory (Ferguson et al., 2001; Choleris et al., 2007). Furthermore, the central amygdala has been associated with the modulation of defensive behavior in the presence of an aversive odor following fear conditioning (Rickenbacher et al., 2017). Finally, the HPA response to predator odor is likely facilitated through main olfactory system-medial amygdala-PVN or olfactory cortex-PVN circuitry (Takahashi, 2014; Kondoh et al., 2016), which would be important to elucidate in the context of PO exposure effects in future studies. Nevertheless, along with the increased PVN *Crf* transcript abundance, our findings suggest that *Oxt* participates in the observed increase social investigation and normal social memory detected in PO offspring.

Within the PVN, PO offspring exhibited increased* Nr3c2* transcript abundance, with no difference in *Nr3c1* transcript abundance. *Nr3c1* and *Nr3c2* expression and function are known to be dissociated, as they respond specifically to low and high level of glucocorticoids, respectively (Brinks et al., 2007; Mesquita et al., 2009; Berardelli et al., 2013; Juruena et al., 2015). There is some evidence that *Nr3c2* is necessary for glucocorticoid regulation of the HPA axis activity during mild stressors, where fewer receptors are associated with a more pronounced corticosterone release. This effect is not observed in response to a stronger stressor (Juruena et al., 2015). This could explain how a change in *Nr3c2* transcript abundance may be sufficient to elicit stress-induced behavioral changes in PO offspring. Within the PVN, *Nr3c2* expression mediates the sensitivity of the stress response through proactive endocrine feedback (Juruena et al., 2015). For example, higher expression of *Nr3c2* promotes resilience, vigilance and selection of the appropriate coping strategy in animals (Juruena et al., 2015; Joëls and de Kloet, 2017). This observation is also in accordance with the behavioral phenotype of increased stress-related behavior and predator odor avoidance previously reported by our group in mouse and rat PO offspring (St-Cyr and McGowan, 2015; St-Cyr et al., 2017). As such, the increased *Nr3c2* transcript abundance in the PVN may contribute to the adaptive stress responsiveness observed in PO offspring.

We demonstrate in this study that a mild and ecologically-relevant prenatal stress is sufficient to elicit changes in stress-induced and social behavior. These results bridge naturalistic and commonly-used laboratory assessments of stress-sensitive behavior, showing a general decrease in exploration and providing evidence that measures in the EPM are potentially informative of naturalistic behaviors. We previously observed differential endocrine responses and transcript abundance in limbic regions in mouse and rat offspring of predator odor exposed dams (St-Cyr and McGowan, 2015; St-Cyr et al., 2017). As the PVN is instrumental in the regulation of endocrine and behavioral responses to stress, the changes observed in transcript abundance observed in this study may constitute part of a unifying mechanism underlying the stress-related behavioral modifications in PO offspring.

## Data Availability

Data supporting the conclusions of the manuscript are available upon request.

## Author Contributions

SS-C and PM designed the study. SS-C and SA conducted the behavioral testing and mice tissue collection. SS-C and RS conducted the qRT-PCR and analyzed the data. PM supervised the research. SS-C, SA, RS and PM wrote the manuscript. All authors contributed to this manuscript and approved the final version of the manuscript.

## Conflict of Interest Statement

The authors declare that the research was conducted in the absence of any commercial or financial relationships that could be construed as a potential conflict of interest. The reviewer MO and handling Editor declared their shared affiliation.

## Footnotes

^1^www.ncbi.nlm.nih.gov

^2^www.useast.ensembl.org

## Abbreviations

MDTB: Mouse defense test battery
PO: Offspring of predator odor exposed dams
PN: Postnatal day
SCT: Social choice test
SIT: Social interaction test.
